# Locomotion selectively enhances visual speed encoding in mouse medial higher visual areas

**DOI:** 10.1016/j.isci.2025.114395

**Published:** 2025-12-09

**Authors:** Edward A.B. Horrocks, Aman B. Saleem

**Affiliations:** 1 Institute of Behavioural Neuroscience, University College London, London WC1V 0AP, UK

**Keywords:** Health sciences

## Abstract

Mammalian visual systems are comprised of multiple brain areas with distinct functional roles. While functional specializations have been proposed in the mouse based on visual feature encoding, the extent to which these specializations are contingent on ongoing behavior is unknown. To address this, we analyzed the neural encoding of visual motion stimuli by thousands of neurons recorded in six cortical and two thalamic visual areas while mice were stationary or locomoting. We found locomotion selectively enhanced visual speed encoding in medial higher visual cortical areas, indicating that these areas may be specialized for processing visual motion during locomotion. By contrast, the encoding of drifting gratings direction was enhanced non-selectively across the mouse visual cortex during locomotion. Our results reveal how a complex interplay of sensory input and ongoing behavior differentially shapes the efficacy of sensory processing in mouse higher visual areas, supporting context-dependent functional roles.

## Highlights

• We characterized visual speed encoding in 1000 s of neurons in 8 mouse visual areas
• Dot field visual speed tuning properties varied between visual areas
• Locomotion selectively enhanced visual speed encoding in medial higher visual areas
• In contrast, locomotion non-selectively enhanced drifting grating direction encoding

## Graphical abstract

**Figure:**
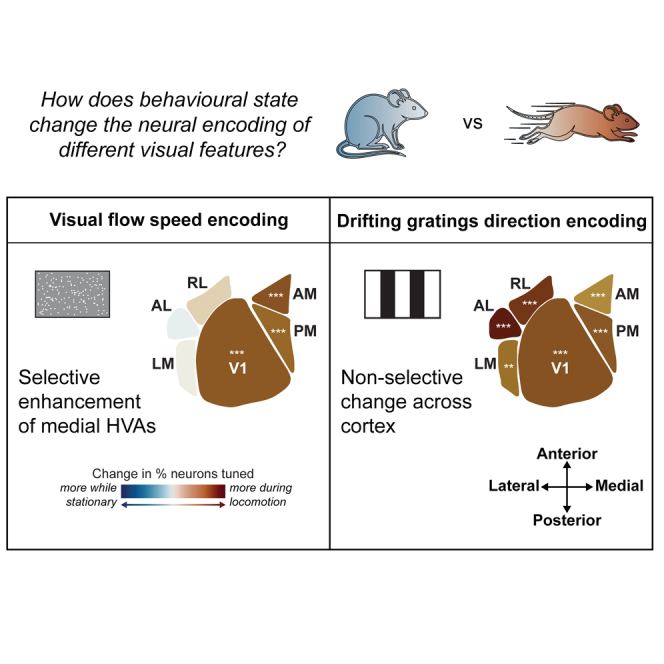

## Introduction

Mammalian visual systems are comprised of multiple brain areas that are specialized for different functional roles.^1^^,^^2^ In primates, two parallel cortical visual processing streams have been identified - a ventral stream associated with object recognition and discrimination and a dorsal stream associated with encoding the spatial location and visual motion of objects, as well as enabling visually guided actions such as locomotion, through, for example, the encoding of optic flow.^3^^,^^4^^,^^5^^,^^6^^,^^7^ Analogous dorsal and ventral cortical processing streams have been proposed in the mouse visual system on the basis of anatomical connectivity^8^^,^^9^^,^^10^^,^^11^ and differences in response properties to visual stimuli.^12^^,^^13^^,^^14^^,^^15^^,^^16^^,^^17^^,^^18^ A particularly striking feature of mouse higher visual areas is their biased representations of the visual field, with lateral areas biased to the central visual field and medial areas to the periphery.^19^^,^^20^ These biases have led to the hypothesis that lateral higher visual areas may be specialized for the encoding of visual landmarks while medial higher visual areas are specialized for the encoding of visual self-motion signals such as optic flow.^21^

Sensory processing in the mouse is strongly modulated by behavior.^22^^,^^23^^,^^24^ In particular, locomotion has striking effects on visual processing, including enhancing the encoding of visual speed in mouse primary visual cortex.^25^ A key outstanding question is whether such behaviour-dependent effects on sensory processing vary between cortical visual areas. Selective enhancement of the encoding of specific visual features during different behaviors could provide important clues about functional specializations of mouse cortical visual areas. Notably, while neurons in different mouse cortical visual areas exhibit varying distributions of tuning properties for locomotion speed^26^^,^^27^^,^^28^^,^^29^ the limited available evidence suggests that locomotion influences visual feature encoding similarly,^24^ perhaps reflecting a general cortex-wide increase in the efficacy of visual encoding. Here, we investigated whether visual flow speed encoding, a feature associated with dorsal-stream processing, is also enhanced globally across mouse visual cortex during locomotion or whether it is selectively enhanced in specific mouse visual areas.

We leveraged large-scale *in vivo* extracellular electrophysiological recordings of thousands of neurons in six cortical and two thalamic mouse visual areas (Allen Institute for Brain Science “Visual Coding” dataset^30^^,^^31^) to analyze the encoding of dot field visual speed during stationary and locomotion behavioral states. We first characterized visual speed tuning properties and found that they differed substantially between visual areas. Broad ranges of tuning properties in V1 and thalamic areas were consistent with functional roles distributing varied information about visual motion throughout the mouse visual system. Narrower ranges of tuning properties in higher cortical visual areas were suggestive of more specialized roles in the processing of visual motion, with a posterior-to-anterior gradient of faster preferred visual speeds reflecting the visual field biases of mouse higher visual areas. When we compared visual speed encoding between behavioral states we found that locomotion enhanced visual speed encoding selectively in medial higher visual areas AM and PM. In contrast, we found a non-selective enhancement of drifting gratings direction encoding across the mouse visual cortex. These findings suggest that medial higher visual areas, which are biased to process the peripheral visual field, may be specialized for the encoding of locomotion-related optic flow. More generally, our findings reveal how a complex interplay of sensory input and ongoing behavior differentially shapes the efficacy of sensory processing in mouse higher visual areas.

## Results

**Figure 1:**
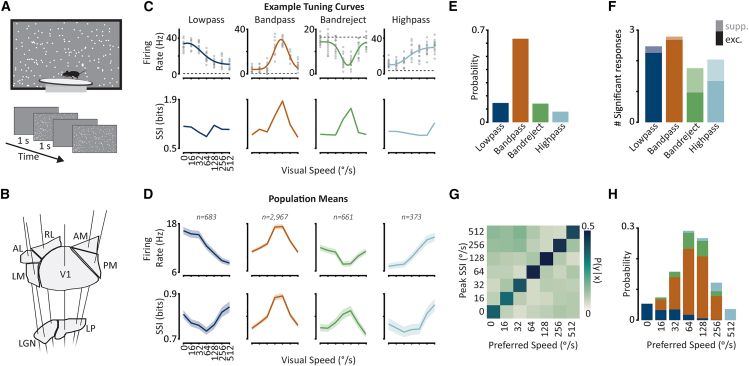
Four classes of visual speed tuning curve across the mouse visual system (A) Schematic of experimental setup. Mice were presented with trials of moving random dot field stimuli whilst free to locomote on a circular treadmill. (B) Schematic of neuropixel probe insertions - six probes simultaneously targeted six cortical and two thalamic visual areas. (C) Examples of four different tuning curve classes from four different neurons (Lowpass unit ID: 951005818, Bandpass: 951002872, Bandreject: 951167265, Highpass: 950997203). *Top row*: Example tuning curves. Each gray circle is a single-trial spike count. Thicker lines are Gaussian descriptive function fits. Dashed lines indicate mean pre-stimulus baseline firing rates. *Bottom row*: Stimulus-specific information (SSI) for the corresponding examples in the top row. See also Figures S2 and S3. (D) Same as (A) for population means of each tuning class. Shaded regions indicate the mean ±95% confidence intervals. Numbers above plots indicate the number of classified tuning curves within each class. (E) Probability histogram of tuning class for all reliable tuning curves. (F) Stacked bar chart showing the number of significant responses for each tuning class (sign-rank test of evoked firing rate vs. baseline firing rate, α < 0.05, Holm-Bonferroni corrected for multiple comparisons). Bars are split into excitatory (dark colors) and suppressed (light colors) responses, based on the difference from activity during pre-stimulus baseline periods. (G) Discrete conditional probability distribution of P(Peak SSI | Preferred Speed) showing correspondence between the two measures. (H) Probability histograms of preferred visual speed for all tuning curves. Stacked bars are color coded according to the classified tuning shape as in (C–F).

We investigated the encoding of visual speed across the mouse visual system during different behavioral states by analysing the firing rate responses of thousands of neurons (*n* = 5,707) to moving dot field stimuli while mice were in stationary or locomoting states (*n* = 19 mice, Figure 1A).^30^^,^^31^ We classified trials as stationary if trial-mean locomotion speed was <0.5 cm/s and remained under 3 cm/s for 75% of the trial. We classified trials as locomotion if trial-mean wheel speed was >3 cm/s and remained over 0.5 cm/s for 75% of the trial (see Figure S1 for distributions of locomotion speeds). We have previously shown this to be a robust criterion for defining the locomotor state of trials.^25^ In this dataset, individual mice tended to either locomote or remain stationary within the stimulus block we analyzed. Six cortical visual areas (V1, LM, AL, RL, AM, PM) and two thalamic visual areas (LGN, LP) were simultaneously targeted in each recording using 6 neuropixel probes (Figure 1B). Stimuli consisted of fields of white dots which covered a large proportion of the contralateral visual field (120° azimuth and 95° elevation) and moved at one of the seven visual speeds (0, 16, 32, 64, 128, 256, 512°/s). We refer to single-neuron firing rate responses as a function of these visual speeds as visual speed tuning, in contrast to the spatial frequency-invariant speed tuning commonly described in response to drifting gratings stimuli.

### Four classes of visual speed tuning across the mouse visual system

We found four classes of visual speed tuning curves in the mouse visual system. To characterize the shapes of tuning curves from neurons across the six cortical and two thalamic areas recorded, we initially performed an unsupervised hierarchical sorting procedure on all reliable tuning curves (*n* = 4,684; Figure S2), where reliability was assessed using the cross-validated coefficient of determination.^25^ This revealed that visual speed tuning curves could broadly be classified into four distinct shapes: lowpass, bandpass, bandreject, and highpass). To characterize the encoding properties of these different tuning classes we performed a model-based classification^26^^,^^27^^,^^29^ of each reliable tuning curve based on the best-fitting of four representative descriptive template functions (Figures 1C and 1D; STAR Methods). The majority of tuned cells exhibited bandpass filtering properties for the visual speeds presented (63%; Figure 1E). Lowpass and bandreject were the next most frequent tuning shapes (lowpass: 15%, bandreject: 14%), followed by highpass (8%).

Excitation and suppression differentially shape visual speed tuning curve classes. To determine whether the different tuning curve shapes were related to excitation, suppression, or a mixture of both, we compared the stimulus responses to pre-stimulus baseline activity levels. There was a significant association between tuning class and the proportions of excitatory and suppressive responses (*p* < 0.001; Kruskal-Wallis test). Bandpass and lowpass tuning curves were shaped primarily by excitatory responses that were either selective or varied systematically as a function of the visual speeds presented (Figures 1F, S3A, and S3B). By contrast, highpass and bandreject tuning curves were shaped by a more even mixture of excitation and suppression (Figures 1F, and S3C, S3D). Interestingly, we found bandreject and highpass tuning curves could result solely from excitatory responses, solely from suppressive responses, or from a mixture of both (Figures S3C and S3D). Suppressive response profiles are reminiscent of “suppressed-by-contrast” cells previously reported in the retina,^32^ LGN^33^ and V1.^34^ Therefore, we demonstrate that selective suppression is a prominent component of visual speed tuning across the mouse visual system, including in higher cortical visual areas.

Different tuning curve classes preferentially encode different visual speeds. To characterize how well different visual speeds are encoded by different tuning curve classes, we used stimulus-specific information (SSI), a mutual information^35^ measure that quantifies how much information is present in responses to a specific stimulus.^36^ For example, a neuron that only responds above baseline levels to one stimulus will have high SSI for that stimulus and low SSI for all others. SSI tended to be positively correlated with spike counts for bandpass and highpass tuning classes and negatively correlated for the bandreject class (Figures 1C, 1D, and S3B–S3D). For lowpass tuning curves, mean SSI peaked for both the slowest and fastest speeds, i.e., the stimuli that evoked the largest and smallest spike count responses (Figures 1C and 1D). This resulted from lowpass tuning curves with peak SSI for the slowest visual speeds, tuning curves with peak SSI for the fastest visual speeds, and tuning curves with peaks for both slow and fast visual speeds (Figure S3A). Overall, visual speeds that evoked the maximum SSI response corresponded reasonably well with the preferred visual speed of a neuron (Figures 1G, 1H, and S3). Thus, low-pass tuning curves best encode slow and fast visual speeds, bandpass and bandreject tuning curves best encode intermediate visual speeds, and high-pass tuning curves best encode fast visual speeds. These tuning classes, therefore, form a complementary coding scheme that encodes the full range of visual speeds presented.

**Figure 2:**
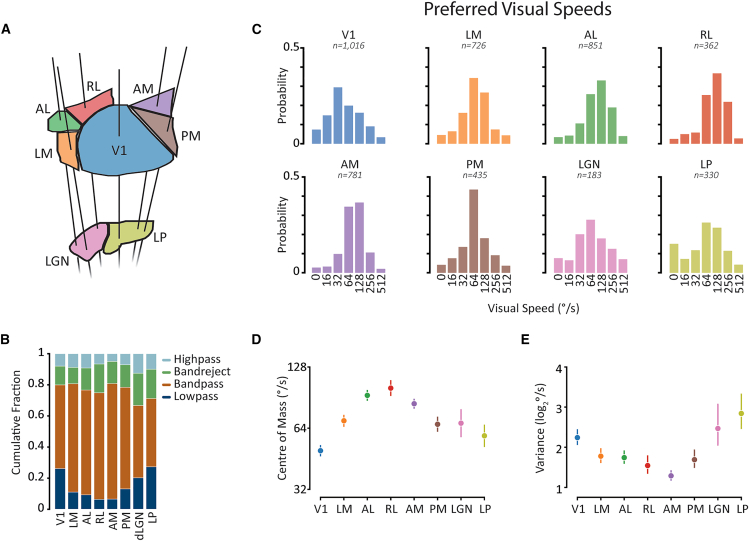
Visual speed tuning properties vary between mouse visual areas (A) Schematic of 8 mouse visual areas (*top* - cortex, *bottom* - thalamus) illustrating the color code applied to all figure panels. (B) Distributions of tuning class for each visual area. (C) Distributions of preferred visual speeds for each visual area. Numbers above plots indicate the number of contributing tuning curves. (D) Center of mass of preferred visual speeds distributions for each brain area (error bars indicate means and 95% confidence intervals). (E) Same as D for the variance of preferred visual speeds distributions. See also Figure S4.

Distributions of visual speed tuning classes vary between mouse visual areas. Having characterized visual speed tuning across the mouse visual system, we next investigated how it varies between areas (Figures 2A and 2B). While bandpass tuning was the most common class of tuning curve in each visual area (range: 44–74%), the overall distribution of tuning classes varied between areas (*p* < 0.001; χ^2^ test, see Figure S4A for pairwise comparisons). For example, in V1 and LP, ∼26% of tuning curves were classified as lowpass, whereas this dropped to ∼6% in RL and AM, with other areas exhibiting intermediate proportions of lowpass neurons. Interestingly, bandreject tuning comprised the second most common tuning class in areas AL (14%), RL (19%), AM (14%), PM (14%), and LGN (21%), demonstrating that this tuning curve shape is widespread in the mouse visual system.

Distributions of preferred visual speeds vary between mouse visual areas. All higher visual cortical areas had distributions shifted to faster preferred visual speeds compared to V1. (Figures 2C and 2D; all *p* < 0.001 LME model followed by pairwise *F*-tests with Holm-Bonferroni correction for multiple comparisons; Figure S3A). Among higher visual areas, there was an anterior-to-posterior gradient of fast-to-slow speed preferences, with anterior areas AL, RL, and AM having the fastest speed preferences and LM and PM the slowest (Figure S4B). Thalamic nuclei had speed preferences intermediate between V1 and higher visual cortical areas. The variance of preferred visual speed distributions also varied between visual areas (*p* < 10^−22^ Levene’s test; Figures 2E and S4C). V1 and thalamic nuclei LGN and LP had broad distributions of visual speed preferences, consistent with the idea that projection neurons from these areas convey diverse information about visual speed to other visual areas.^10^,^28^^,^^37^^,^^38^^,^^39^ Higher visual cortical areas, in contrast, had more concentrated distributions of visual speed preferences, suggesting that these areas may be specialized for encoding specific ranges of visual speeds.

### Locomotion selectively enhances visual speed tuning in medial higher visual areas as well as primary visual cortex and thalamus

**Figure 3:**
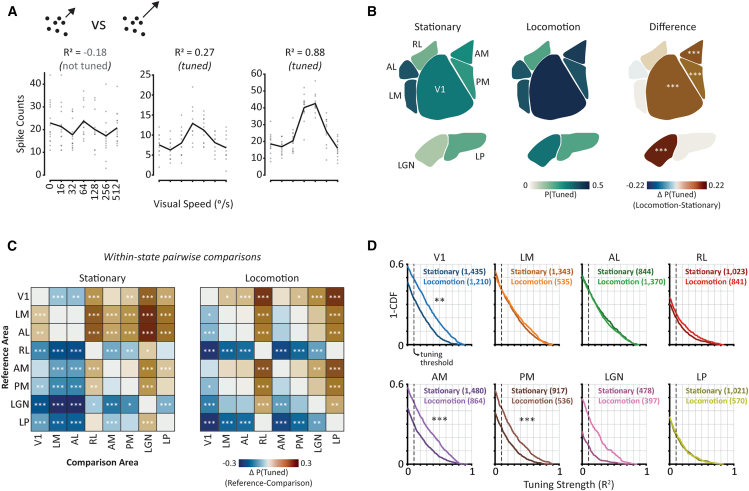
Locomotion selectively enhances visual speed tuning in medial higher visual areas AM and PM, as well as V1 and LGN (A) Example tuning curves with different tuning strength (R^2^) values. Circles indicate individual trial spike counts and black lines the stimulus-conditioned mean of those spike counts. Inset icon illustrates a stimulus consisting of moving dot fields. (B) Probability of tuning for visual speed by visual area during stationary states (*left*), locomotion states (*center*), and the difference between locomotion and stationary states (*right*). *p*-values are calculated using post-hoc *F-*tests on generalized linear mixed effects (GLME) model weights to test for the effect of state on the probability of being tuned in each visual area, with Holm-Bonferroni multiple comparisons correction. (C) Pairwise comparisons of P(tuned) between visual areas during stationary (left) and locomotion (right) states. *p*-values are calculated using post-hoc *F-*tests on GLME model weights for each visual area within a state with Holm-Bonferroni correction for multiple comparisons. (D) Distributions of tuning strength for each visual area, separately for stationary (dark colors) and locomotion (light colors) states. The dashed vertical line indicates the threshold used to determine tuning. Numbers in brackets indicate the number of contributing tuning curves. *p*-values are calculated using post-hoc *F-*tests on linear mixed effects (LME) model weights to compare the effect of state in each visual area and are corrected for multiple comparisons using Holm-Bonferroni correction. See also Figures S5 and S6. ∗*p* < 0.05, ∗∗*p* < 0.01, ∗∗∗*p* < 0.001 (with Holm-Bonferroni multiple comparisons correction).

Tuning for visual features can be strongly modulated by behavioral state in the mouse visual system.^24^^,^^40^^,^^41^ We therefore investigated whether the prevalence and strength of visual speed tuning varied between behavioral states in cortical and thalamic areas of the mouse visual system. We assessed the strength of tuning using the cross-validated coefficient of determination method^25^ (R^2^; Figure 3A), a metric that determines how reliable a tuning curve is across repeated trials compared to a flat mean firing rate model. We classified a neuron to be tuned for visual speed if its tuning strength was both significant (assessed using a shuffled distribution of trial spike counts) and greater than a threshold (R^2^ ≥ 0.1). We obtained similar results regardless of the specific tuning strength threshold we used (Figure S5). Tuning strength is positively correlated with both a previously used measure of tuning reliability^29^ and mutual information (Figure S6). We used mixed-effects models to test whether differences between behavioral states and visual areas were significant, as they allowed us to account for various random factors such as subject (see STAR Methods).

The prevalence of visual speed tuning varied between visual areas during stationary states (Figures 3B and 3C). Lateral higher visual areas AL and LM had the highest prevalence of visual speed-tuned cells in the stationary state (Figures 3B and 3C; LM: 38%, AL: 39%; *p* < 0.01 compared to all other visual areas; GLME model followed by pairwise *F*-tests with Holm-Bonferroni corrections for multiple comparisons), followed by V1 (31%). Medial higher visual area AM and PM had the next highest prevalence of visual speed tuning (AM: 27%, PM: 25%) followed by LP, RL, and LGN (LP: 20%, RL: 18%, LGN: 12%). Thus, the prevalence of tuning tended to be lower in thalamic areas compared to cortex, except that LP was higher than RL (Figures 3B and 3C). The weak tuning for visual speed in LGN suggests that V1 either integrates weakly tuned feedforward inputs from LGN or that recurrent cortical circuits strengthen visual speed tuning in V1 during this state.

Locomotion selectively increased the prevalence of tuning for visual speed in medial higher visual areas AM and PM, as well as V1 and LGN. When comparing the prevalence of visual speed tuning between stationary and locomotion states, we found clear area-dependent effects. Specifically, during locomotion, there was a significant increase in the prevalence of visual speed tuning in medial higher visual areas AM (Figure 3B; *p < 0.001*, 27% versus 43%) and PM (*p < 0.001*, 25% vs. 39%). By contrast, differences in lateral higher visual areas were small and not statistically significant (Figure 3B). We also found a significant increase in the proportion of neurons tuned for visual speed during locomotion in V1 (*p* < 0.001, 31% vs. 47%), in agreement with our previous findings,^25^ as well as in LGN (*p* < 0.001, 12% vs. 32%), but not in LP.

Analysis of tuning strength values similarly revealed a selective enhancement of visual speed tuning in medial higher visual areas during locomotion. Distributions of tuning strength shifted toward higher values for AM (*p < 0.001*, LME model analysis) and PM (*p < 0.001*), but not for lateral higher visual areas during locomotion (Figure 3D). We also observed an increase in the strength of tuning for neurons in V1 during locomotion (*p < 0.01*). The increase in tuning strength in LGN did not reach statistical significance after correcting for multiple comparisons (corrected *p* = 0.16). When we restricted our analysis to cells that passed the tuning strength threshold, we also found a significant increase in tuning strength for V1 (mean ± SEM in stationary states: 0.35 ± 0.01 locomotion: 0.43 ± 0.01, *p* = 0.002), AM (stationary: 0.36 ± 0.01, locomotion: 0.42 ± 0.01, *p* = 0.002), PM (stationary: 0.33 ± 0.01, locomotion: 0.40 ± 0.01, *p* = 0 < 0.001) and LGN (stationary: 0.26 ± 0.02, locomotion: 0.37 ± 0.02, *p* = 0.047). As a result of these changes, AM and V1 had the strongest tuning for visual speed during locomotion, compared to AL and LM during stationary states (Figure S5).

### Locomotion selectively enhances visual speed population decoding in medial higher visual areas as well as primary visual cortex and thalamus

**Figure 4:**
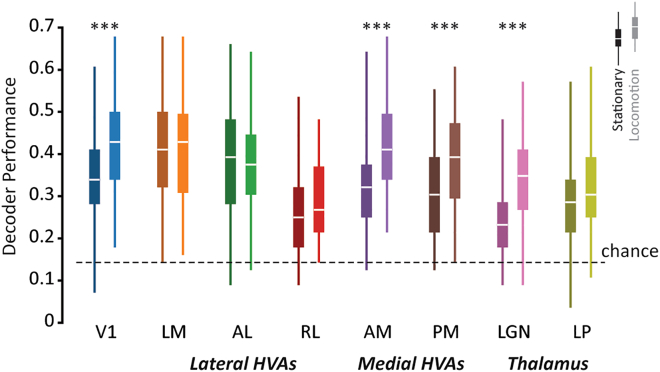
Locomotion selectively enhances visual speed population decoding in medial higher visual areas AM and PM, as well as V1 and LGN Poisson independent decoder (PID) decoding performance (fraction of correctly predicted held out test trials) of subsampled populations (*n* = 10) in each visual area during stationary (darker left-side boxplots) and locomoting (lighter right-side boxplots) states. Boxplots indicate full distributions (boxes are interquartile range, white lines are medians). *∗∗∗p < 0.001* (Holm-bonferroni-corrected) post-hoc *F*-tests on LME model weights to test for the effect of state on decoding performance, for each visual area.

We next investigated how locomotion affects visual speed decoding at a population level. Stimulus decoding provides a measure of how much stimulus information is contained within the responses of populations of neurons. We therefore compared the decoding performance of populations of neurons between behavioral states for each visual area (Figure 4), using a Poisson Independent Decoder (PID).^42^^,^^43^^,^^44^ Because different numbers of neurons were simultaneously recorded in each visual area in each subject, we tested small populations of simultaneously recorded neurons (*n* = 10) at a time to facilitate a comparison of the different visual areas and behavioral states.

We found that locomotion was associated with the enhanced decoding of visual speed in medial higher visual areas AM (mean ± SEM performance during stationary states: 0.32 ± 0.01, locomotion states: 0.41 ± 0.01, *p < 0.001* effect of state, LME model analysis) and PM (stationary: 0.30 ± 0.01, locomotion: 0.38 ± 0.02, *p < 0.001*) as well as V1 (stationary: 0.34 ± 0.01, locomotion: 0.43 ± 0.01, *p < 0.001*) and LGN (stationary: 0.24 ± 0.01, locomotion: 0.32 ± 0.01, *p < 0.001*), in agreement with our findings that visual speed tuning is improved in these areas. By contrast, we found no significant difference in decoding performance between states for the lateral higher visual areas (LM, AL and RL) or the thalamic nucleus LP (all *p > 0.05*). Thus, the area-specific changes in visual speed tuning we observed during locomotion were associated with corresponding area-specific changes in the ability to decode visual speed from neural population activity.

### Locomotion non-selectively enhances drifting gratings' direction tuning across the mouse visual cortex

**Figure 5:**
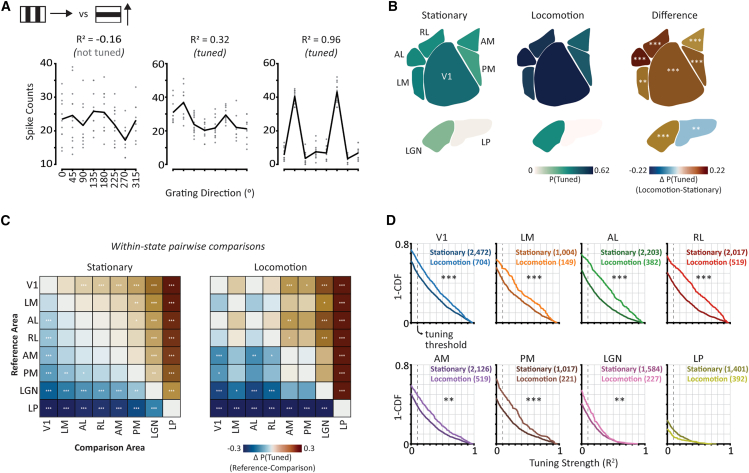
Locomotion non-selectively enhances drifting gratings direction tuning across the mouse visual cortex (A) Example tuning curves with different tuning strength (R^2^) values. Circles indicate individual trial spike counts, and black lines the stimulus condition mean of those spike counts. Inset icon illustrates a stimulus consisting of drifting gratings moving in different directions. (B) Probability of tuning for drifting grating direction by visual area during stationary states (*left*), locomotion states (*center*), and the difference between locomotion and stationary states (*right*). *p*-values are calculated using post-hoc *F-*tests on generalized linear mixed effects (GLME) model weights to test for the effect of state on the probability of being tuned in each visual area, with Holm-Bonferroni multiple comparisons correction. (C) Pairwise comparisons of P(tuned) between visual areas during stationary (left) and locomotion (right) states. *p*-values are calculated using post-hoc *F-*tests on GLME model weights for each visual area within a state with Holm-Bonferroni correction for multiple comparisons. (D) Distributions of tuning strength for each visual area, separately for stationary (dark colors) and locomotion (light colors) states. The dashed vertical line indicates the threshold used to determine tuning. Numbers in brackets indicate the number of contributing tuning curves. *p*-values are calculated using post-hoc *F-*tests on linear mixed effects (LME) model weights to compare the effect of state in each visual area and are corrected for multiple comparisons using Holm-Bonferroni correction. ∗*p* < 0.05, ∗∗*p* < 0.01, ∗∗∗*p* < 0.001 (with Holm-Bonferroni multiple comparisons correction).

Does the selective enhancement of medial higher visual areas AM and PM during locomotion reflect general changes in visual processing across visual areas, or are these changes stimulus-specific? To address this question, we repeated our visual feature tuning analysis for drifting gratings direction in stationary and locomotion states (Figure 5) using a related dataset from the Allen Institute (“*Brain Observatory 1.1”* stimulus set^30^). The experimental protocols were identical to those analyzed above except that a different set of stimuli was presented.

Locomotion non-selectively enhanced drifting gratings direction tuning across all cortical areas. Unlike visual speed encoding, there was no selective change in the encoding of drifting gratings direction during locomotion (Figures 5B–5D). Instead, both the prevalence and strength of direction tuning increased for all visual areas during locomotion, except for LP. Indeed, the largest increases in the prevalence of drifting gratings direction tuning were in lateral visual areas AL and RL, in contrast to our findings of selective increases in the prevalence of visual speed tuning in medial higher visual areas AM and PM. Notably, neurons in area RL were robustly tuned for drifting gratings direction in both behavioral states (Figure 5), in contrast to our findings of weak visual speed tuning (Figure 3), indicating that moving dot fields are ill-suited to driving neurons in area RL (see also^45^). Our results establish that the effects of behavioral state on sensory processing in the mouse visual system exhibit a complex brain area-dependence that is contingent on the specific sensory feature being encoded.

## Discussion

We have shown that the effects of behavioral state on sensory processing differ between mouse higher visual areas, providing evidence that their functional roles are contingent on ongoing behavior. By comparing the responses of thousands of neurons recorded across six cortical and two thalamic visual areas, we found that the encoding of visual flow speed is selectively enhanced in medial higher visual areas during locomotion (Figure 3). This is in direct contrast to the non-selective enhancement of drifting gratings direction encoding that occurs globally across mouse visual cortex during locomotion (Figure 5; see also^29^). Our findings establish that during locomotion, there is not simply a cortex-wide increase in the efficacy of visual encoding, but instead that locomotion-dependent changes in visual processing vary non-trivially between visual areas, dependent on the specific visual feature being encoded.

Why are improvements in visual speed encoding during locomotion specific to medial higher visual areas AM and PM? A prominent feature of mouse higher visual areas is their biased representations of the visual field.^19^ These biases have been hypothesized to reflect distinct functional specializations during active navigation.^21^ Medial higher visual areas AM and PM have representations biased to the peripheral visual field, leading to the suggestion that they may be specialized for processing optic flow during self-motion.^21^ Consider that during forward locomotion, with a focus of expansion directly ahead, optic flow vectors in the periphery are generally largest and therefore likely to be most informative about many aspects of self-motion. Indeed, human perceptual studies indicate that peripheral vision may play a particularly important role in the visual estimation of forward self-motion speed.^46^^,^^47^^,^^48^ If a similar reliance on the periphery for estimating forward self-motion speed is present in mice, then the selective enhancement of visual speed encoding in mouse medial higher visual areas may serve to improve this perceptual estimation during locomotion. Why the mouse visual system exhibits area-selective changes in the encoding of some visual features but non-selective changes in the encoding of others remains an intriguing open question.

Supporting evidence for the hypothesis that AM and PM are important for optic flow processing during self-motion is that they have been identified anatomically as mouse dorsal stream areas with connectivity to motor and navigation-related areas.^9^ Indeed, AM and PM are located between the primary visual cortex and the retrosplenial cortex, the latter an area considered important for visuospatial processing during navigation and with strong connections to the hippocampus. AM and PM also exhibit large receptive field sizes and weak surround suppression,^30^^,^^49^ features suitable for encoding wide-field visual motion such as optic flow. These features are reminiscent of the primate dorsal stream area MST, which is specialized for encoding optic flow. Neurons in area MST have large receptive field sizes, preferentially receive projections from populations that represent the peripheral visual field and can be modulated by non-visual self-motion signals.^50^^,^^51^^,^^52^ The enhanced encoding of the visual speed in AM and PM during locomotion may therefore reflect a functional specialization for visually-guided self-motion estimation.

The different visual field biases in higher visual areas may also explain their different visual speed tuning properties. We observed an anatomical gradient of average preferred visual speeds, with neurons in anterior higher visual areas AL, RL, and AM preferring the fastest visual speeds overall (Figures 2C and 2D). Anterior higher visual areas are biased toward lower elevations of the visual field,^19^ which may naturally expose them to faster visual speeds due to the proximity of the floor plane in mice. The variance of preferred visual speed distributions also differed substantially between areas (Figures 2C and 2E), with broad distributions of preferred visual speeds in V1 and thalamic areas LGN and LP. These broad distributions are consistent with these areas distributing varied visual motion information throughout the mouse visual cortex.^18^^,^^28^ The narrower distributions in higher visual cortical areas, which were concentrated at intermediate to fast visual speeds, suggest more specialized roles in processing specific visual speeds.

Our results provide new insights into the mouse higher visual area PM, whose functional role has been particularly ambiguous. While anatomically located within the mouse dorsal stream,^9^ the spatiotemporal tuning properties of PM neurons in response to drifting gratings stimuli are more ventral stream-like, i.e., selectivity for high spatial frequency and low temporal frequency.^12^^,^^13^^,^^16^ Indeed, in agreement with a more ventral stream-like role, we found that visual speed encoding was poor in PM in stationary states (Figures 3B, 3C, and 4). However, during locomotion, we observed a large, significant enhancement of visual speed encoding, a feature that one might associate with dorsal-stream visual processing. Additionally, our finding that PM neurons tend to exhibit faster visual speed preferences than V1 appears, at first glance, to contrast with previous work that reported that PM neurons preferred slow-moving gratings.^12^ This discrepancy may instead reflect robust stimulus-dependent tuning properties of neurons in the mouse visual system. While PM neurons may be tuned to high spatial and low temporal frequencies for spatially localized grating stimuli, our analysis reveals that for wide-field, moving dot field stimuli, they are tuned to faster speeds than V1 neurons. This striking stimulus-dependence is consistent with previous work showing that the visual tuning properties of mouse V1 neurons can differ substantially between drifting gratings and visual flow stimuli.^53^ Other experimental differences, for example, Ca^2+^ imaging in^12^ compared to extracellular electrophysiology here, may also contribute to differences in findings.

The bias for superior visual speed encoding in lateral higher visual areas in stationary mice (Figure 3C) is unexpected. Whilst area AL has been characterized as a dorsal stream area^9^ and has been previously shown to perform well encoding the motion direction of random dot kinematograms similar to those used here,^17^ area LM is often considered a ventral stream area with properties more suitable for visual texture discrimination.^17^^,^^54^ This lateral bias is not a general principle of visual processing since there was no significant difference in the prevalence of drifting gratings tuning between higher visual cortical areas during stationary states (Figure 5C). Nor was this lateral bias for visual speed encoding present during locomotion, but instead replaced by a more even distribution of visual speed encoding across the mouse visual cortex (Figure 3B). One possibility is that strong visual speed encoding in lateral higher visual areas serves object motion perception during stationary states.

In conclusion, our results reveal important insights into the functional roles of mouse higher visual cortical areas by demonstrating that the effects of behavior are both brain area and stimulus-dependent. These findings emphasize a complexity of function that is context-dependent, promoting the importance of considering behavior, and context more generally, when determining functional specializations of mouse visual areas. As such, future experimentation taking into account ongoing behavior and task context will be important for elaborating the functional roles mouse visual areas serve in visual perception and visually-guided action.

### Limitations of the study

A limitation of our study is that we were unable to perform detailed within-neuron comparisons of visual speed tuning during stationary and locomotion states. This was because individual subjects tended to either locomote or remain stationary for the majority of the stimulus blocks we analyzed, coupled with the modest number of trial repeats per stimulus condition. Additionally, while a large number of cells were analyzed from each visual area, recordings were consistently targeted at the retinotopic center of each area^55^ and were biased to record more cells from cortical layer 5. Thus, it is possible that the populations of cells recorded and analyzed do not fully capture the properties of the visual areas in which they are located. Where comparisons are available, our results are in agreement with prior work - our findings replicate a previous study^29^ showing that the encoding of drifting gratings direction encoding is enhanced across the mouse visual cortex during locomotion. We also previously reported enhanced visual speed encoding in mouse V1 during locomotion.^25^

## Resource availability

### Lead contact

Requests for further information and resources should be directed to and will be fulfilled by the lead contact, Edward A. B. Horrocks (edward.horrocks.17@ucl.ac.uk).

### Materials availability

This study did not produce any new unique reagents.

### Data and code availability

• We analyzed an open-access dataset from the Allen Institute for Brain Science.^30^^,^^31^ We provide minimally preprocessed data and analysis code to make the reproduction of our results easier.
• Minimally processed data have been deposited at Figshare (https://doi.org/10.5522/04/30136174.v1).
• All original code is available on GitHub (https://github.com/eabhorrocks/HorrocksSaleem_AllenVisSpeed) and has been deposited at Zenodo (https://doi.org/10.5281/zenodo.17512798).

## Acknowledgments

This work was supported by The Sir Henry Dale Fellowship from the 10.13039/100010269Wellcome Trust and 10.13039/501100000288Royal Society (200501); the Human Frontier in Science Program (RGY0076/2018), 10.13039/501100000268Biotechnology and Biological Sciences Research Council grant (R004765 and BB/W01579X/1), UKRI Frontier Research grants (EP/Y024656/1) to A.B.S.; and 10.13039/501100000268Biotechnology and Biological Sciences Research Council studentship to E.H. We thank Sam Solomon for comments on the article.

## Author contributions

This work was conceptualized by E.H. and A.B.S.; methodology, software, and formal analysis were by E.H.; visualization and writing were by E.H. and A.B.S.; and supervision and funding acquisition were by A.B.S.

## Declaration of interests

The authors declare no competing interests.

## Declaration of generative AI and AI-assisted technologies in the writing process

During the preparation of this work, the authors used Gemini (Google) and ChatGPT (OpenAI) large language models in order to improve clarity of the text. After using this tool/service, the authors reviewed and edited the content as needed and take full responsibility for the content of the publication.

## STAR★Methods

### Key resources table

**Table:** 

REAGENT or RESOURCE	SOURCE	IDENTIFIER
**Deposited data**		

Allen Brain Observatory - Visual Coding - Neuropixels Dataset	Allen Institute for Brain Science	brain-map.org/explore/circuits/visual-coding-neuropixels
Processed data Alen Brain Observatory data	This paper; Figshare	https://doi.org/10.5522/04/30136174.v1

**Experimental models: Organisms/strains**		

C57BL/6J mice	Jackson Laboratories	N/A
Pvalb-IRES-Cre × Ai32 mice	Allen Institute for Brain Science	N/A
Sst-IRES-Cre × Ai32 mice	Allen Institute for Brain Science	N/A
Vip-IRES-Cre × Ai32 mice	Allen Institute for Brain Science	N/A

**Software and algorithms**		

MATLAB 2023a	MathWorks	N/A
Custom analysis code	Github & Zenodo	https://github.com/eabhorrocks/HorrocksSaleem_AllenVisSpeed (https://doi.org/10.5281/zenodo.17512798)

### Experimental model and subject details

We analyzed a large-scale *in-vivo* extracellular electrophysiology dataset (*Visual Coding - Neuropixels*^30^^,^^31^) made open access by the Allen Institute. We analyzed two datasets with separate stimulus sets. Mice were maintained in the Allen Institute for Brain Science animal facility and used in accordance with protocols approved by the Allen Institute’s Institutional Animal Care and Use Committee. The experiments were covered by IACUC protocol #1805 at the time they were performed. Analysis of visual speed encoding used the *Functional Connectivity* stimulus set, and analysis of drifting gratings direction encoding used the *Brain Observatory 1.1* stimulus set. Experimental protocols were identical for the two stimulus sets except for the visual stimuli presented. A full overview of the experimental subjects included for analysis is available in Table S1. We provide a summary below:

We analyzed 12 *Functional Connectivity* sessions with sufficient stationary trials (*n* = 7 male, *n* = 5 female; age 114–142 days; *n* = 8 wild-type C57BL/6J, *n* = 3 Sst-IRES-Cre × Ai32, *n* = 1 Vip-IRES-Cre × Ai32) and 8 *Functional Connectivity* sessions with sufficient locomotion trials (*n* = 5 male, *n* = 3 female; age 108–135 days; *n* = 4 wild-type C57BL/6J, *n* = 2 Sst-IRES-Cre × Ai32, *n* = 2 Vip-IRES-Cre × Ai32).

We analyzed 16 *Brain Observatory 1.1* sessions with sufficient stationary trials (*n* = 13 male, *n* = 3 female; age 98–140 days; *n* = 9 wild-type C57BL/6J, *n* = 4 Pvalb-IRES-Cre × Ai32, *n* = 3 Vip-IRES-Cre × Ai32) and 6 *Brain Observatory 1.1* sessions with sufficient locomotion trials (*n* = 4 male, *n* = 2 female; age 93–122 days; *n* = 1 wild-type C57BL/6J, *n* = 4 x Sst-IRES-Cre × Ai32, *n* = 1 Vip-IRES-Cre × Ai32).

We did not include sex in our study design as it was unlikely to be relevant to our scientific findings. Additionally, there was insufficient statistical power to analyze the effects of sex on our results.

### Method details

#### *In vivo* electrophysiology

In each recording session (one per mouse) six single-shank neuropixel probes were acutely inserted to record extracellularly in six cortical visual areas: primary visual cortex (V1; Allen CCFv3 acronym *‘VISp’*), lateromedial area (LM; *‘VISl’*), anterolateral area (AL; *‘VISal’*), rostrolateral area (RL; *‘VISrl’*), anteromedial area (AM; *‘VISam’*) and posteromedial area (PM; *‘VISpm’*), and two thalamic areas: dorsal lateral geniculate nucleus (LGN; *'dLGN'*) and lateral posterior nucleus (LP; *'LP'*). Each neuropixel probe targeted the retinotopic center (i.e., along the optic axis of the contralateral eye) of each visual cortical area, based on a previously generated retinotopic map obtained using intrinsic signal imaging. During each session, mice were head-fixed and free to locomote on a circular treadmill whilst passively viewing stimuli, of which we analyzed a subset.

#### Visual stimuli

To investigate the encoding of visual speed, we analyzed neural responses to moving dot field stimuli (‘Functional Connectivity’ visual stimulus set). Stimuli consisted of fields of ∼200 white moving dots (diameter = 3°) which covered a large proportion of the contralateral visual field (120° azimuth and 95° elevation). On each trial, dots moved at one of the seven visual speeds (speeds = 0, 16, 32, 64, 128, 256, 512°/s) in one of 4 directions (0°, 45°, 90°, 135°, where 0° = left-to-right), with 90% coherence. Stimulus duration was 1s with a 1s gray screen inter-stimulus interval. Because many cells in the mouse visual system exhibit direction-selectivity, and speed tuning could vary as a function of direction of motion, we analyzed each direction of motion independently. To control for any potential effects of direction of motion on our results we included direction as a random factor in our statistical analyses (see below).

We also analyzed the encoding of drifting grating direction (‘Brain Observatory 1.1’ visual stimulus set). Full-screen sinusoidal drifting gratings (spatial frequency = 0.04 cycles/°, contrast = 80%) moved in one of the 8 directions (0–315°, equally spaced) at one of the 5 temporal frequencies (1, 2, 4, 8, 15Hz). Stimulus duration was 2s with a 1s gray screen inter-stimulus interval. As with our analysis of speed tuning for moving dot fields, we analyzed tuning for drifting gratings direction independently for each temporal frequency. To control for any potential effects of temporal frequency we included it as a random factor in our statistical analyses (see below).

#### Spike-sorting

Data were spike-sorted using the Allen Institute’s in-house spike sorting pipeline^30^ which uses Kilosort2^56^ to perform initial spike sorting followed by a number of custom post-processing modules that remove double-counted spikes and noise units and compute a number of waveform and cluster quality metrics.

We restricted our analysis to ‘good’ clusters, which we defined based on 3 criteria^25^^,^^57^: 1) Refractory period violations ≤10%; 2) Amplitude distribution cut-off ≤10%; and 3) Mean amplitude ≥50μV.

Based on these criteria we analyzed 4,500 units in the *Functional Connectivit*y dataset (V1, stat: 443, run: 347; LM, stat: 359, run: 190; AL, stat: 271, run: 387; RL, stat: 282, run: 269; AM, stat: 445, run: 303; PM, stat: 279, run: 170; LGN, stat: 139, run: 121, LP, stat: 294, run: 201) and 4,712 units in the *Brain Observatory 1.1* dataset (V1, stat: 639, run: 226; LM, stat: 290, run: 58; AL, stat: 566, run: 139; RL, stat: 489, run: 195; AM, stat: 548, run: 189; PM, stat: 275, run: 94; LGN, stat: 377, run: 111, LP, stat: 333, run: 183).

### Quantification and statistical analyses

Statistical parameters such as *n* values, measures of central tendency and measures of dispersion are reported in the main text and figure legends. Details of all statistical tests can be found in Table S2.

#### Classification of trials according to behavioral state

We classified the behavioral state of trials (defined as the stimulus duration epoch) according to the locomotion speed of mice recorded by a rotary encoder. We classified trials as stationary if trial-mean locomotion speed was <0.5 cm/s and remained under 3 cm/s for 75% of the trial. We classified trials as locomotion if trial-mean wheel speed was >3 cm/s and remained over 0.5 cm/s for 75% of the trial. We have previously shown this to be a robust criteria for defining the locomotor state of trials.^25^

To calculate locomotion speed, we resampled the rotary encoder data at 100Hz and smoothed it using a Gaussian kernel with a standard deviation of 35 ms.

To investigate visual speed tuning we considered each direction of motion independently. To investigate drifting grating direction tuning we considered each temporal frequency independently. We only analyzed responses where there were at least 8 trials for each visual speed/motion direction that were classified with the same behavioral state (stationary or locomotion).

#### Tuning strength

To assess tuning strength we calculated the cross-validated Coefficient of Determination (R^2^) on trial-based spike counts.^25^ To enable a fair comparison we downsampled trial counts to 8 (minimum required for data inclusion).

We implemented 3-fold cross-validation by randomly dividing trials into a training set comprising 2/3 of the trials and a test set comprising the remaining 1/3 (equally sampled from each stimulus condition). During each iteration, two models were created using the training data: a tuning curve model (trained model) representing the mean spike count responses to individual stimulus conditions and a null model representing the average spike count across all stimulus conditions. Using the test data, we constructed a test model by calculating the mean spike count for each stimulus condition.

To evaluate the performance of the trained model and the null model, we calculated the sum-of-squared residuals between each model and the test model. The coefficient of determination (R^2^) was then computed using the following equation:

$$R^{2}=1-\frac{{SS}_{model}}{{SS}_{null}}\;\}\;if\;{SS}_{model\;}\leq {SS}_{null\;}$$

(Equation 1) $$R^{2}=-1+\frac{{SS}_{null}}{{SS}_{model}}\;\}\;if\;{SS}_{model\;}>{SS}_{null\;}$$

where ${SS}_{model}$ is the sum of squared residuals between the *trained model* and the *test model* and ${SS}_{null}$ is the sum of squared residuals between the *null model* and the *test model*.

We computed the mean R^2^ value over the 3 cross-validations, using a unique set of test trials on each iteration. We repeated this entire process 10 times with different random splits of train and test trials, providing 10 estimates of R^2^, and took the final estimate of R^2^ as the mean of these 10 values. To assess the statistical significance of these tuning strength values, we also generated a shuffled distribution of R^2^ values for each neuron by performing the same 3-fold cross-validation procedure on randomly shuffled spike counts, repeated 100 times.

We considered a neuron to be tuned if R^2^ ≥ 0.1 and R^2^ ≥ 95th percentile of the shuffled distribution (*p* ≤ 0.05). For comparisons of tuning strength we set a floor of R^2^ = 0.

#### Tuning curve sorting

To sort tuning curves for visual speed we used an unsupervised hierarchical method. We first determined which tuning curves were reliably ‘tuned’ using the criteria described above. We then generated a dissimilarity matrix by calculating the euclidean distance between pairs of (z-scored) reliable tuning curves. Following this, we obtained an initial dendrogram (MATLAB function *linkage*) with the unweighted average distance. We then found the optimal leaf order using an algorithm that minimises the sum of pairwise distances between neighboring leaves^58^ (MATLAB function *optimalleaforder*).

#### Tuning shape classification

We classified tuning curves using the best-fitting of four descriptive functions (lowpass, bandpass, bandreject and highpass). Descriptive functions were gaussians parameterised as:

(Equation 2) $$f\left(x\right)=b+ae^{\frac{{\left(x-u\right)}^{2}}{2\sigma^{2}}}$$

Where b is a baseline firing rate parameter, a is an amplitude parameter, u is the mean, σ is the standard deviation, and x is the index of the stimulus conditions (i.e., 1:7 for the seven visual speeds presented).

Tuning classes were differentiated using different parameter bounds. Lowpass and highpass were described by two functions each (with negative and positive amplitudes). Each function has an appropriately bounded mean parameter. The upper bound of bandpass and bandreject was limited to ensure well-defined maxima or minima. Additionally, for a tuning curve to be classified as bandpass or bandreject, the fitted function was required to exhibit a peak with a prominence of at least 1/3 of the spike-count range of the tuning curve, else the next best-fitting function was used to classify the tuning shape. We calculated prominence using the MATLAB function *findpeaks*. In the case of a single-peaked Gaussian, the prominence is calculated as follows (paraphrased from MATLAB documentation): 1) Find the peak of the descriptive function; 2) find the minimum of the signal to the left and right of the peak; 3) take the larger of these two values as the reference level; 4) calculate the prominence as the distance between the peak height and the reference level. Bandreject functions were inverted before performing this calculation.

To determine if there was a statistically significant difference in the distributions of tuning shape between visual areas we performed a $\chi^{2}$ test of homogeneity. Pairwise comparisons between areas were Bonferroni-Holm-corrected for multiple comparisons.

#### Preferred visual speed classification

Preferred visual speeds were classified as the visual speed that evoked the maximum mean spike count response for lowpass, bandpass and highpass tuning curves and as the visual speed that evoked the minimum mean spike count response for bandreject tuning curves.

To determine if there was a statistically significant difference in the preferred visual speeds of neurons in different visual areas we used a linear mixed-effects (LME) model with the following equation:

(Equation 3) PreferredVisualSpeed∼Area+(1|Subject)+(1|Direction)+(1|RF)

where PreferredVisualSpeed is the log_2_-transformed preferred visual speed of a tuning curve, Area is a categorical variable representing the area the neuron was recorded from, (1|Subject) is a random intercept that takes into account variation between subjects and (1|Direction) is a random intercept that takes into account variation between different directions of motion. Pairwise comparisons between areas were performed using post-hoc *F*-tests and Bonferroni-Holm-corrected for multiple comparisons.

To determine if there was statistically significant differences in the variance of preferred visual speed distributions between visual areas we used Levene’s test for equality of variance. Pairwise comparisons between areas were Bonferroni-Holm-corrected for multiple comparisons.

#### Stimulus-specific information (SSI) and mutual information

To calculate SSI we first binned responses into 7 quantiles (after downsampling to 8 trials for each stimulus condition) to enable a fair comparison between cells with large differences in firing rates. We then calculated SSI using the following equation from Butts, (2003):

(Equation 4) $$i_{ssi}\left(s\right)=\sum_{r}p(r|s)\left\{H\left[S\right]-H\left[S|r\right]\right\}$$

Where $i_{ssi}\left(s\right)$ is the stimulus-specific information for stimulus *s*, p(r|s) is the conditional probability distribution of responses *r* given stimulus *s*, H[S] is the entropy of the stimulus distribution and H[S|r] is the conditional entropy associated with response *r*. The {H[S]−H[S|r]} term is collectively known as the specific information of a response *r*.

We also computed the total mutual information between a set of spike counts and the stimuli presented, which in the case of equal trial counts is the mean value of SSI across the different stimuli presented.

#### Response excitation and suppression

To determine whether a spike-count response to an individual stimulus condition was excitatory or suppressed we compared it to the baseline firing rate (200 ms before stimulus onset). For each tuning curve we tested whether there was a significant change in firing rate between pre-stimulus and stimulus epochs using the Holm-Bonferroni-corrected Wilcoxon signed-rank test. We then determined responses with significant changes in firing rate as excitatory or suppressed based on the direction of change in firing rate. To test whether there was a statistically significant difference in the proportions of excitatory vs. suppressive response between different tuning classes we calculated the proportion of significant responses that were excitatory for all tuned cells with at least one significant response). We then performed a Kruskal-Wallis test on these values, grouped by tuning class.

#### Statistical analysis of tuning strength

We utilised mixed-effects models for many of our statistical analyses. Since multiple neurons were recorded from each subject, individual measurements are not independent, violating a key assumption of standard linear models. Mixed-effects models resolve this by simultaneously estimating the fixed effects of interest (e.g., differences across brain areas or between behavioral states) and the random variance attributable to grouping factors such as individual subjects. This allowed us to handle unbalanced sample sizes across subjects (i.e., different numbers of neurons were recorded in each brain area in each animal) and preserves the statistical power of the full dataset without resorting to averaging, which could obscure effects at the single-neuron level.

To test whether there were statistically significant differences in the probability of neurons being tuned for visual speed or drifting grating direction between different brain areas and behavioral states we used a generalized linear mixed effects (GLME) model:

(Equation 5) ResponseVariable∼Area:State+(1|Subject)+(1|Direction)+(1|RF)

Where ResponseVariable was P(Tuned), a binomial response variable indicating whether a set of responses were tuned (1) or not (0), Area:State denotes an interaction between brain area and behavioral state, (1|Subject) is a random intercept that takes into account variation between subjects, (1|Direction) is a random intercept that takes into account variation between different directions of motion and (1|RF) is a random intercept that takes into account variation due to the presence or not of a significant receptive field. We found that including random effects did not improve the GLME’s used to assess the probability of tuning as a function of brain area and behavioral state (assessed using likelihood ratio), so we did not include these variables.

To test whether there were statistically significant differences in the tuning strength of neurons between brain areas and behavioral states we used a linear mixed effects (LME) model using Equation 5 with the exception that ResponseVariable term was a continuously valued Tuning Strength variable. In this case random effects did improve the performance of the models.

We used a similar approach to analyze tuning for drifting gratings direction, with the difference that we included temporal frequency as a random factor instead of motion direction.

We performed pairwise *F*-tests on model coefficients to test for statistical significance between different areas and behavioral states. To control the family-wise error rate when performing multiple comparisons we used the Holm-Bonferroni correction. The raw *p*-values were first ordered from smallest to largest. Each *p*-value was then compared to a progressively less stringent alpha level, calculated as α/(m−i+1), where α was set at 0.05, m was the total number of comparisons, and i was the *p*-value’s rank. The procedure ceased at the first instance where a *p*-value exceeded its adjusted alpha level, and all subsequent comparisons were considered non-significant.

#### Receptive field significance

We used pre-computed *p*-values for the significance of receptive fields (see^30^ for details) as provided by the Allen Institute SDK. Briefly, a 2D histogram of spike counts is computed in response to presentations of a 9x9 grid of Gabor stimuli. A chi-square test statistic was then computed as follows:

(Equation 6) $$\chi^{2}=\sum_{i=0}^{n}\frac{{\left(E_{i}-O_{i}\right)}^{2}}{E_{i}}$$

Where $O_{i}$ is the average response of a cell at stimulus location *i* and $E_{i}$ is the expected grand average response per stimulus presentation.

The statistical significance of this Chi-square value was then determined by comparing it against the null distribution of test statistics calculated after shuffling stimulus locations.

#### Decoding analysis

To decode visual speed from the spike counts of neurons we used a Poisson Independent Decoder (PID) that assumes independent neurons.^42^^,^^43^^,^^44^ For each session, populations of 10 neurons were randomly selected without replacement from individual visual areas. Decoding was conducted separately for each direction of motion.

To compare decoding performance across populations recorded in different sessions, we standardized the number of trials used for decoding by limiting the dataset to 8 trials per stimulus condition (8 trials × 7 speeds = 56 trials total). Leave-one-out cross-validation was used, wherein spike counts from all but one trial per condition (7 trials × 7 speeds = 49 trials) were used to train the decoder, and the remaining trials (1 trial × 7 speeds = 7 trials) were used to test it. This process was repeated until all trials were tested. Decoder performance was determined as the proportion of correctly classified test trials.

For each trial, the predicted visual speed was determined by maximizing the following log likelihood function:

(Equation 7) $$logL\left(\theta \right)=\sum_{i=1}^{N}W_{i}\left(\theta \right)r_{i}-B\left(\theta \right)$$

Where *i* indexes over *N* neurons, ${\;W}_{i}\left(\theta \right)$ is the log of the mean spike count of neuron i for a given stimulus θ learned from training data, $r_{i}$ is the number of spikes produced by neuron i on the trial being predicted and B(θ) is a bias correction term calculated as the sum (over N neurons) of mean spike counts for stimulus θ.

To test whether there were statistically significant differences between behavioral states for each brain area we first fit an LME with Equation 5, where the *ResponseVariable* was Decoding Performance. We then performed pairwise *F*-tests on model coefficients (see analysis of tuning above).

## Supplemental information

Document S1. Figures S1–S6

Table S1. Overview of sessions included for analysis

Table S2. Supplementary statistical information
