# Supplementary material for: Locomotion selectively enhances visual speed encoding in mouse medial higher visual areas
Source: iScience. 2025 Dec 9;29(1):114395. doi: 10.1016/j.isci.2025.114395 (PMC12796763; doi:10.1016/j.isci.2025.114395)
Supplement: Document S1. Figures S1–S6 [file mmc1.pdf]

iScience, Volume *29*

Supplemental information

Locomotion selectively enhances visual speed encoding in mouse medial higher visual areas

Edward A.B. Horrocks and Aman B. Saleem

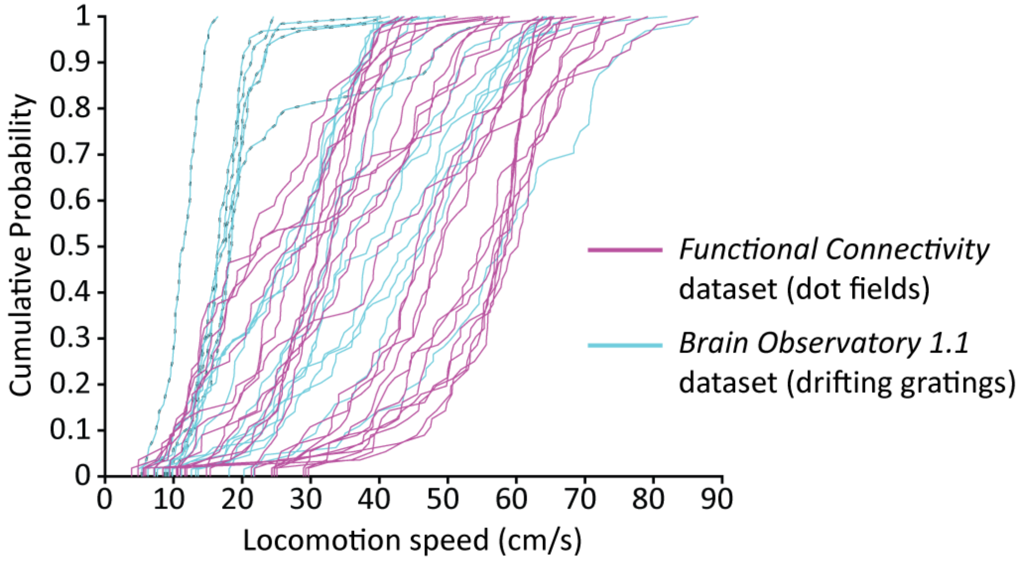

Figure S1: Cumulative distributions of locomotion speed, related to Figures 3, 4 and 5. Cumulative distributions of mean locomotion speed during locomotion trials for dot fields (magenta) and drifting gratings (cyan). Each line represents the distribution of locomotion speeds (averaged over the stimulus period) for a single subject and direction of motion (dot fields) or temporal frequency (drifting gratings). Slower sets of locomotion trials from *Brain Observatory 1.1* dataset are highlighted by a black dashed line. Excluding these trials from our analyses did not qualitatively change our results.

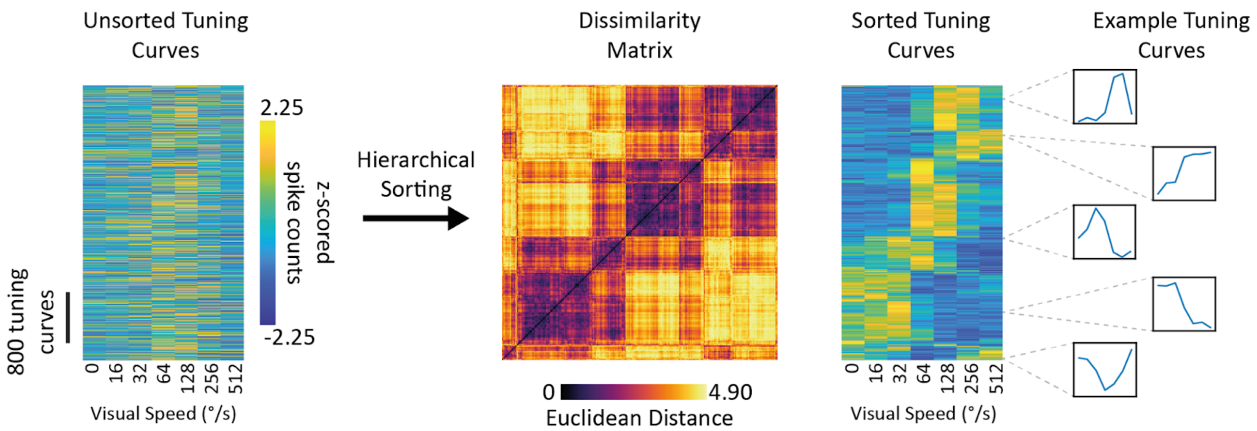

Figure S2: Hierarchical sorting of visual speed tuning curves, related to Figure 1.

Overview of tuning curve sorting procedure. We used hierarchical sorting to investigate different tuning curve shapes within the mouse visual system. *Left*: Unsorted tuning curves (z-scored). *Middle left*: dissimilarity matrix after sorting. *Middle right*: Sorted tuning curves. *Right:* representative example tuning curves. Dashed lines indicate location in the sorted tuning curves plot.

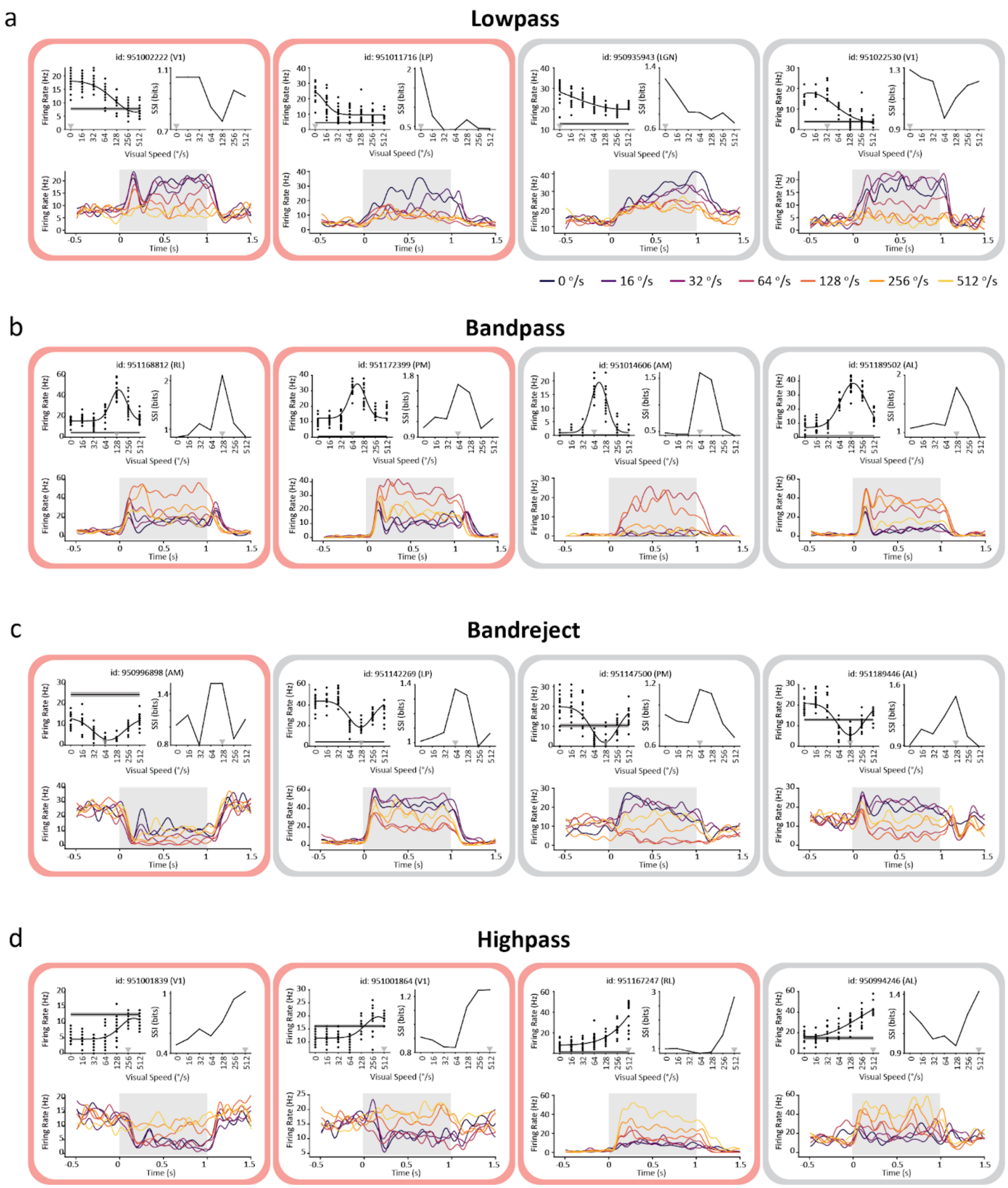

Figure S3: Example tuning curves for each tuning curve class, related to Figure 1.
a Four example lowpass tuning curves. *Top left:* tuning curves. Each grey circle is a single-trial spike count. Thicker lines are gaussian descriptive function fits. Shaded lines indicate mean pre-stimulus baseline firing rates. The preferred speed is indicated by a triangle on the x-axis. *Top right:* corresponding SSI curve. The speed that evoked the maximum SSI response is indicated by a triangle on the x-axis. *Bottom*: Peri-stimulus time histograms of smoothed firing rates (gaussian kernel with σ = 35ms). Shaded

grey region indicates stimulus period. Shaded outline of example indicates behavioural state in which tuning curve was recorded in (stationary in grey, locomotion in red). **b-d** same as a for bandpass (b), bandreject (c) and highpass (d) tuning curves.

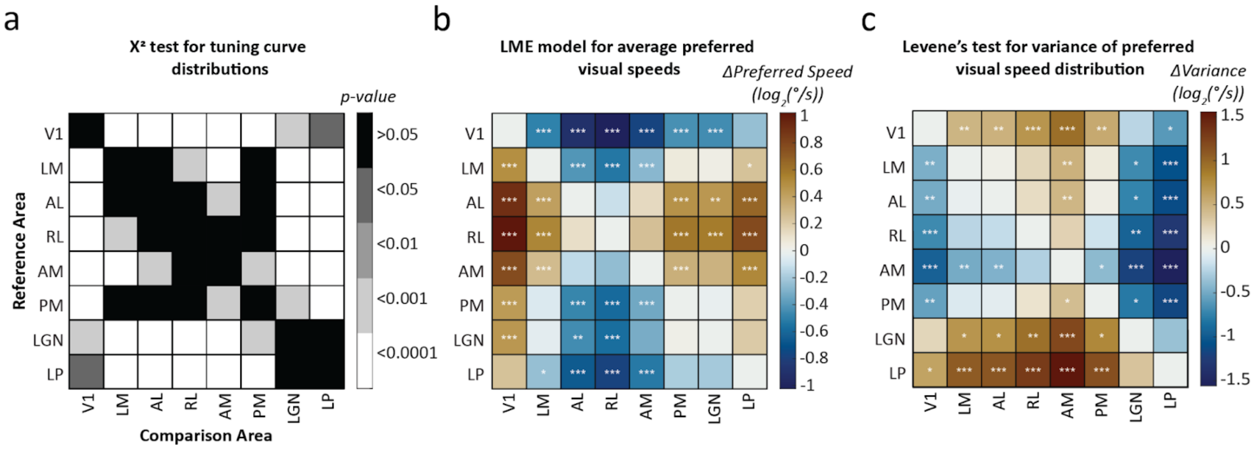

Figure S4: Pairwise statistical analyses between visual areas for tuning curve characterisations, related to Figure 2.

a) *p*-values from X^2 tests for distributions of tuning shape (n = 1,016 (V1), 726 (LM), 851 (AL), 362 (RL), 781 (AM), 435 (PM), 183 (LGN), 330 (LP) tuning curves). Holm-Bonferroni correction for multiple comparisons. **b)** Differences in centre of mass of preferred visual speed distributions between areas, significance tested using LME model followed by pairwise *F*-tests between areas. n-values and correction for multiple comparisons as in (a). **c)** Differences in variance of preferred visual speed distributions, significance tested using Levene's test for equality of variance. n-values and corrections for multiple comparisons as in (a).

* p<0.05, ** p<0.01, *** p<0.001 (with Holm-Bonferroni multiple comparisons correction).

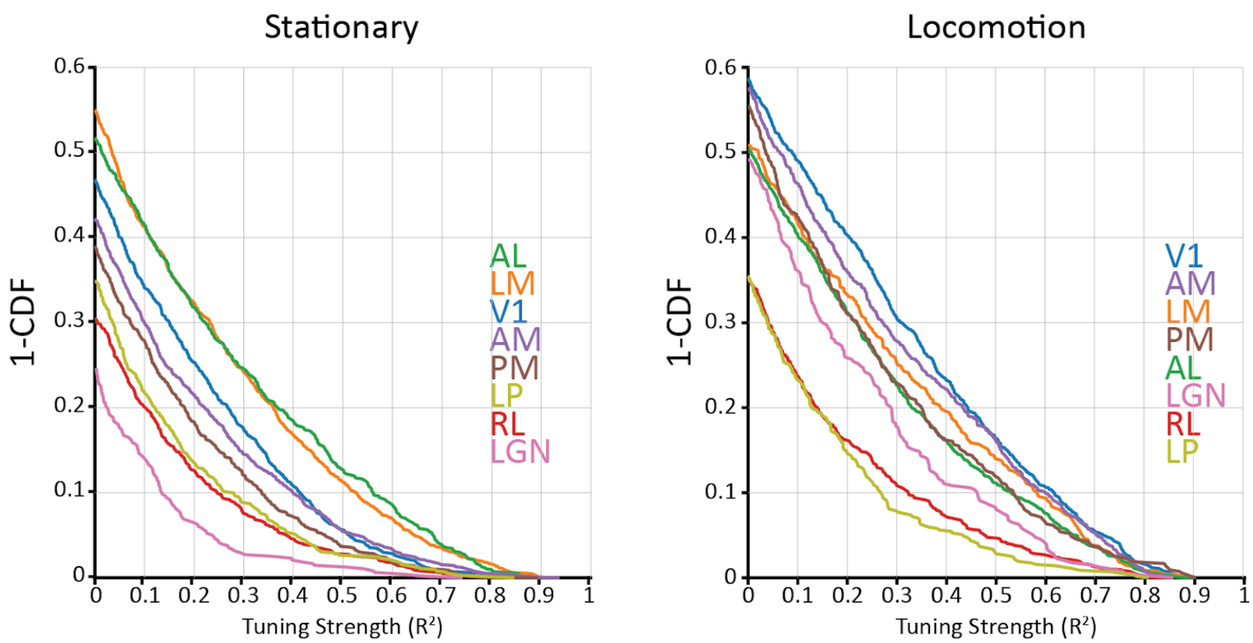

Figure S5: Distributions of tuning strength for visual areas during stationary and locomotion states, related to Figure 3.
Survival functions (1 - cumulative distribution function) of tuning strength for each visual area during stationary (left) and locomotion (right) states.

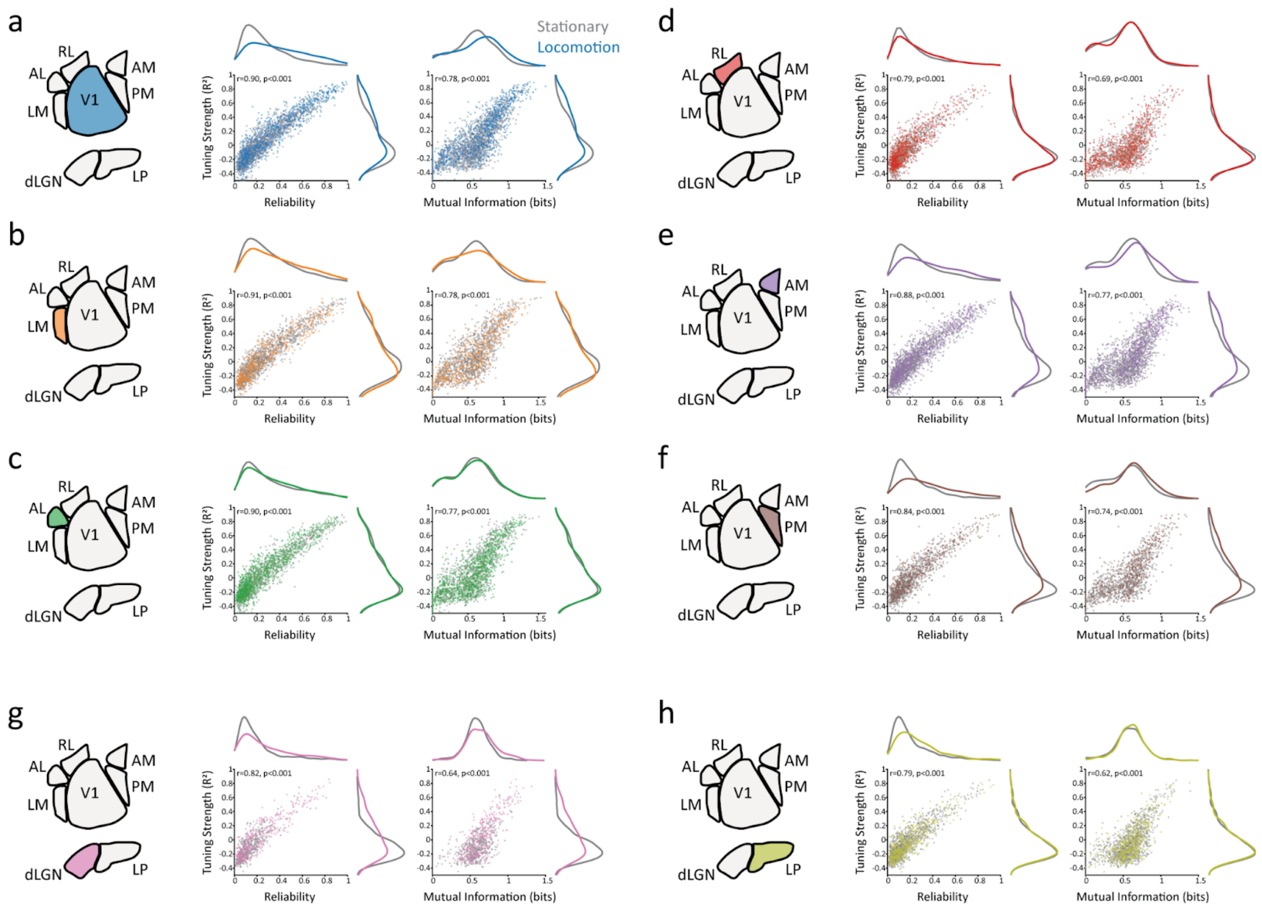

Figure S6: Relationship between tuning strength and other metrics, related to Figure 3.
a) *Left:* Schematic of all cortical and thalamic visual areas analysed with V1 highlighted. *Centre:* Relationship between tuning strength and tuning 'reliability' (variance of the mean spike count tuning curve as a function of visual speed divided by the variance of all spike counts) for V1 neurons. Individual data points represent individual tuning curves from stationary (grey) and locomotion (coloured) states. Marginal histograms (smoothed) are plotted along the corresponding axes for the two quantities. The rank correlation between these variables and corresponding p-value are noted. *Right:* same as centre for tuning strength and mutual information. **b-h)** Same as **a** for all other visual areas analysed.
